# Corvis ST Assessment of Corneal Biomechanical Properties in Patients with Type 2 Diabetes Mellitus

**DOI:** 10.3390/medicina62101928

**Published:** 2026-10-07

**Authors:** Aida Kasumović Bećirević, Armin Kasumović, Ines Matoc Kasumović, Ivan Merdžo, Ana Kovač, Marija Veselinović, Mirko Jankov, Sanja Sefić Kasumović

**Affiliations:** 1 Department of Ophthalmology, Private Eye Clinic Dr. Sefić, Ferhadija 5, 71000 Sarajevo, Bosnia and Herzegovina; kasumovic.armin@gmail.com (A.K.); inesmatoc@gmail.com (I.M.K.); ana.kovac@stu.ssst.edu.ba (A.K.); info@sefic.ba (S.S.K.); 2 Department of Ophthalmology, University Hospital Mostar, University of Mostar School of Medicine, 88000 Mostar, Bosnia and Herzegovina; ivan.merdzo@mef.sum.ba; 3 Department of Ophthalmology, University Clinical Center Niš, 18000 Niš, Serbia; drmarija.veselinovic@gmail.com; 4 Centre for Microsurgery LaserFocus, 11000 Belgrade, Serbia; mirkojankov@mac.com; 5 Institut für Refraktive und Ophthalmo-Chirurgie, 8200 Zurich, Switzerland

**Keywords:** corneal biomechanics, diabetes mellitus, Corvis ST, corneal deformability, deformation amplitude

## Abstract

*Background and Objectives*: Diabetes mellitus (DM) is associated with structural and biochemical changes in the cornea that may influence its biomechanical behavior. This study aimed to compare corneal biomechanical properties between healthy individuals and patients with type 2 DM. *Materials and Methods*: This observational cross-sectional study was conducted at a specialized ophthalmology center from September 2025 to April 2026 and included 115 eyes, which were divided into two groups: 60 healthy control eyes and 55 eyes of patients with DM type 2 (DM2 group). All subjects underwent a complete ophthalmological examination and corneal biomechanical screening using Corvis ST (Oculus Optikgeräte GmbH, Wetzlar, Germany). The study included participants aged 50–70 years, with central corneal thickness from 500 to 600 μm, and normal ocular findings. Exclusion criteria included a history of any previous corneal disease, uveitis, glaucoma, spherical refractive errors exceeding ±3.00 D, cylindrical refractive errors exceeding 3.00 D, prior ocular surgery, contact lens wear and systemic connective tissue diseases. *Results*: DM2 group was older than the control group (median age 63 years [IQR 57–68] vs. 56 years [IQR 54–59], *p* < 0.001). In unadjusted comparisons, deformation amplitude at highest concavity (DA HC) was higher in the DM2 group (1.05 [0.98–1.09] vs. 0.98 [0.91–1.06] mm, *p* = 0.002), as was integrated radius (IR) (8.00 [7.53–8.40] vs. 7.50 [6.60–8.60] mm^−1^, *p* = 0.032), whereas radius of curvature at highest concavity (RC HC) (7.41 [6.77–7.86] vs. 7.52 [7.10–8.04] mm, *p* = 0.038) and stiffness parameter at first applanation (SP-A1) (115.50 [102.18–128.40] vs. 122.50 [107.90–142.90] mmHg/mm, *p* = 0.023) were lower. After adjustment for age, gender, central corneal thickness and intraocular pressure, only DA HC remained independently associated with DM2 status (β = 0.033 mm, 95% CI 0.007–0.058; *p* = 0.012). *Conclusions*: Patients with type 2 DM exhibited a corneal biomechanical profile characterized by greater corneal deformability, reflected by higher DA HC and IR values and lower RC HC and SP-A1 values in unadjusted comparisons. Of these parameters, increased DA HC remained independently associated with DM2 after multivariable adjustment. These findings highlight the complexity of diabetes-related corneal biomechanical alterations and suggest that they may not uniformly manifest as increased corneal stiffness.

## 1. Introduction

Diabetes mellitus (DM) represents one of the most serious global health problems and a major cause of mortality worldwide. According to the International Diabetes Federation, an estimated 589 million adults aged 20–79 years were living with diabetes worldwide in 2024, and this number is projected to increase to approximately 853 million by 2050 [1]. Chronic hyperglycemia increases the risk of developing both microvascular and macrovascular complications, which remain the major contributors to morbidity and mortality in patients with diabetes [2,3,4]. Diabetes may affect multiple ocular structures, and although diabetic retinopathy remains one of the most clinically significant and extensively studied ocular complications, increasing evidence suggests that diabetes also induces significant alterations of the cornea. Corneal abnormalities, collectively referred to as diabetic keratopathy, have been reported in up to 70% of patients with diabetes, yet these changes remain frequently underdiagnosed because clinical attention is predominantly focused on retinal disease [2,5,6]. Clinically, diabetes-related corneal changes include increased central corneal thickness, epithelial fragility, recurrent erosions, persistent epithelial defects, delayed wound healing, endothelial dysfunction, reduced corneal sensitivity, and dry eye disease. These alterations are thought to result from epithelial and endothelial dysfunction, oxidative stress, extracellular matrix remodeling, accumulation of advanced glycation end products (AGEs), and increased collagen cross-linking. Such mechanisms may alter the biomechanical properties of the cornea and contribute to diabetic keratopathy [6,7,8].

Corneal biomechanics has emerged as an important component of corneal physiology and plays a significant role in the diagnosis and management of various ocular disorders, including glaucoma, keratoconus, systemic diseases such as diabetes mellitus, and in assessment for refractive surgery [7,9,10]. The cornea is a viscoelastic tissue whose biomechanical properties are primarily determined by the organization of collagen fibrils and the extracellular matrix (ECM) within the stroma. The ECM, composed of collagen, proteoglycans, glycoproteins, and other macromolecules, provides structural support, maintains corneal transparency, and contributes to wound healing and mechanical stability. Corneal biomechanics can be influenced by several factors, including ECM composition, hydration, environmental factors (e.g., eye rubbing, smoking, and ultraviolet exposure), and hormonal changes [9,10,11]. Modern non-contact imaging technologies enable in vivo assessment of the corneal response to a standardized air puff, providing objective biomechanical parameters that may improve our understanding of diabetes-related corneal changes. Among these technologies, the Corvis ST (Oculus Optikgeräte GmbH, Wetzlar, Germany) combines dynamic Scheimpflug imaging with high-speed analysis of corneal deformation and provides multiple biomechanical indices [7,9,10].

Although several studies have investigated the relationship between diabetes mellitus and corneal biomechanics, the available evidence remains inconsistent, and it is still unclear whether diabetes results in clinically significant alterations of corneal biomechanical properties. Moreover, studies evaluating diabetic corneas using Corvis ST remain relatively limited [7,10,11].

Therefore, the aim of this study was to compare corneal biomechanical properties between healthy individuals and patients with type 2 diabetes mellitus using the Corvis ST and to evaluate whether disease duration is associated with alterations in corneal biomechanical parameters.

## 2. Materials and Methods

This observational cross-sectional study was conducted from September 2025 to April 2026 at the Eye Polyclinic “Dr Sefić”, Sarajevo. A total of 115 participants were enrolled, with only one eye per participant included in the analysis. The study population was divided into two groups: a control group comprising 60 eyes from participants without a known diagnosis of diabetes mellitus and a DM group comprising 55 eyes from patients with DM type 2 (DM2 group). All subjects underwent a complete ophthalmological examination, including the best corrected visual acuity, slit-lamp biomicroscopy, Goldmann applanation tonometry and fundoscopy. For the purposes of the present study, normal ocular findings were defined as the absence of clinically relevant ocular pathology on ophthalmological examination, including corneal disease, uveitis, glaucoma, clinically significant lens opacity, retinal or macular disease, and other ocular conditions that could potentially affect the assessed corneal biomechanical parameters. In participants with DM, the presence and severity of diabetic retinopathy (DR) were assessed by dilated fundus examination and classified according to the International Clinical Diabetic Retinopathy Disease Severity Scale proposed by Wilkinson et al. [12]. DR severity was categorized as no apparent DR, mild non-proliferative diabetic retinopathy (NPDR), moderate NPDR, severe NPDR, or proliferative diabetic retinopathy (PDR). Grading was performed as part of the routine ophthalmological examination. All participants underwent corneal tomography using the Pentacam (Oculus, Wetzlar, Germany) and corneal biomechanics using the Corvis ST (Oculus Optikgeräte GmbH, Wetzlar, Germany), with software version 6.12r87. A single Corvis ST measurement was initially obtained for each eye. Measurements were repeated when necessary to obtain an “OK” quality status. When multiple acceptable scans were available, the single best-quality scan was selected for analysis. All the measurements were made between AM 10:00 and PM 15:00. The study followed the tenets of the Declaration of Helsinki, and all protocols were approved by the Ethics Committee of the Polyclinic. Informed consent was obtained from all subjects involved in the study.

The inclusion criteria for the study were both male and female participants aged 50–70 years, with central corneal thickness (CCT) from 500 to 600 μm, and normal ocular findings (no ocular pathology expected to affect corneal biomechanical measurements). For inclusion in the control group, participants were required to have no known history of diabetes mellitus based on medical history and available medical records. Fasting plasma glucose and HbA1c were not systematically measured as part of the study protocol in the control group. Exclusion criteria included a history of any previous corneal disease, uveitis, glaucoma, prior intraocular lens (IOL) implantation or other ocular surgery, prior contact lens wear and systemic connective tissue diseases. To minimize the potential influence of refractive status on corneal biomechanical parameters, subjects with spherical refractive errors exceeding ±3.00 D and/or cylindrical refractive errors exceeding 3.00 D were also excluded [13,14].

The following Corvis ST parameters were analyzed: CCT, Stress–Strain Index (SSI), Corvis Biomechanical Index (CBI), biomechanical glaucoma factor (BGF), length of flattened cornea at the first applanation (A1L), velocity at the first applanation (A1V) and time taken to reach the first applanation (A1T); length of flattened cornea at the second applanation (A2L), velocity at the second applanation (A2V) and time taken to reach the second applanation (A2T); radius of curvature of the cornea at highest concavity (RC HC), peak distance at maximal concavity (PD), deformation amplitude at highest concavity (DA HC), non-contact tonometer measured IOP (IOPnct), biomechanically corrected intraocular pressure (bIOP), deformation amplitude ratio (DA ratio), integrated radius (IR), Ambrósio relational thickness to the horizontal profile (ARTh) and stiffness parameter at first applanation (SP-A1).

Statistical analysis was performed using IBM SPSS Statistics software version 32.0 (IBM Corp., Armonk, NY, USA). Data distribution was assessed using the Shapiro–Wilk test. As the variables were not normally distributed, continuous data were presented as median values with interquartile range (IQR). Differences in corneal biomechanical parameters were compared using the Mann–Whitney U test. For between-group comparisons, effect estimates were expressed as Hodges–Lehmann median differences with corresponding 95% confidence intervals. Multivariable linear regression analyses were additionally performed to assess whether group status was independently associated with the main biomechanical parameters after adjustment for potential confounding factors. Age, sex, CCT, and intraocular pressure were included as covariates in all models. Regression coefficients (β) with 95% confidence intervals (CIs) and *p*-values were reported. The normality of residuals was assessed for each model. Correlations between disease duration and corneal biomechanical parameters were assessed using Spearman’s rank correlation coefficient (ρ), with corresponding 95% confidence intervals. Statistical significance was defined as *p*-value < 0.05. ChatGPT version 4o was used for grammar checking and language editing of this manuscript.

## 3. Results

This study included 115 patients with ages ranging from 50 to 70 years. The sample included 60 healthy eyes (control group) and 55 eyes of patients with type 2 DM. Median age in the control group was 56 (with IQR 54–59), and in DM2 group was 63 (IQR 57–68). DM2 group included 31 male (56.4%) and 24 female (43.6%) participants, while control group included 23 male (38.3%) and 37 female (61.7%) participants. Among diabetic patients 39 (70.9%) were treated with oral antidiabetic drugs, while 16 (29.1%) were receiving insulin therapy. Diabetic retinopathy was present in 24 patients (43.6%), while 31 patients (56.4%) had no evidence of diabetic retinopathy. Baseline demographic data are shown in Table 1.

### 3.1. Pachymetry Values and Corneal Biomechanical Parameters

In the current study, CCT, SSI, CBI, BGF, A1L, A1V, A1T, A2L, A2V, A2T, PD, IOPnct, bIOP, DA ratio and ARTh did not show statistically significant difference between healthy eyes and eyes of patients with DM. On the other hand, statistical significance was shown for the following parameters: DA HC, RC HC, IR and SP-A1. Unadjusted comparisons of pachymetric and corneal biomechanical parameters between the DM2 and control groups are presented in Table 2.

**Figure 1:**
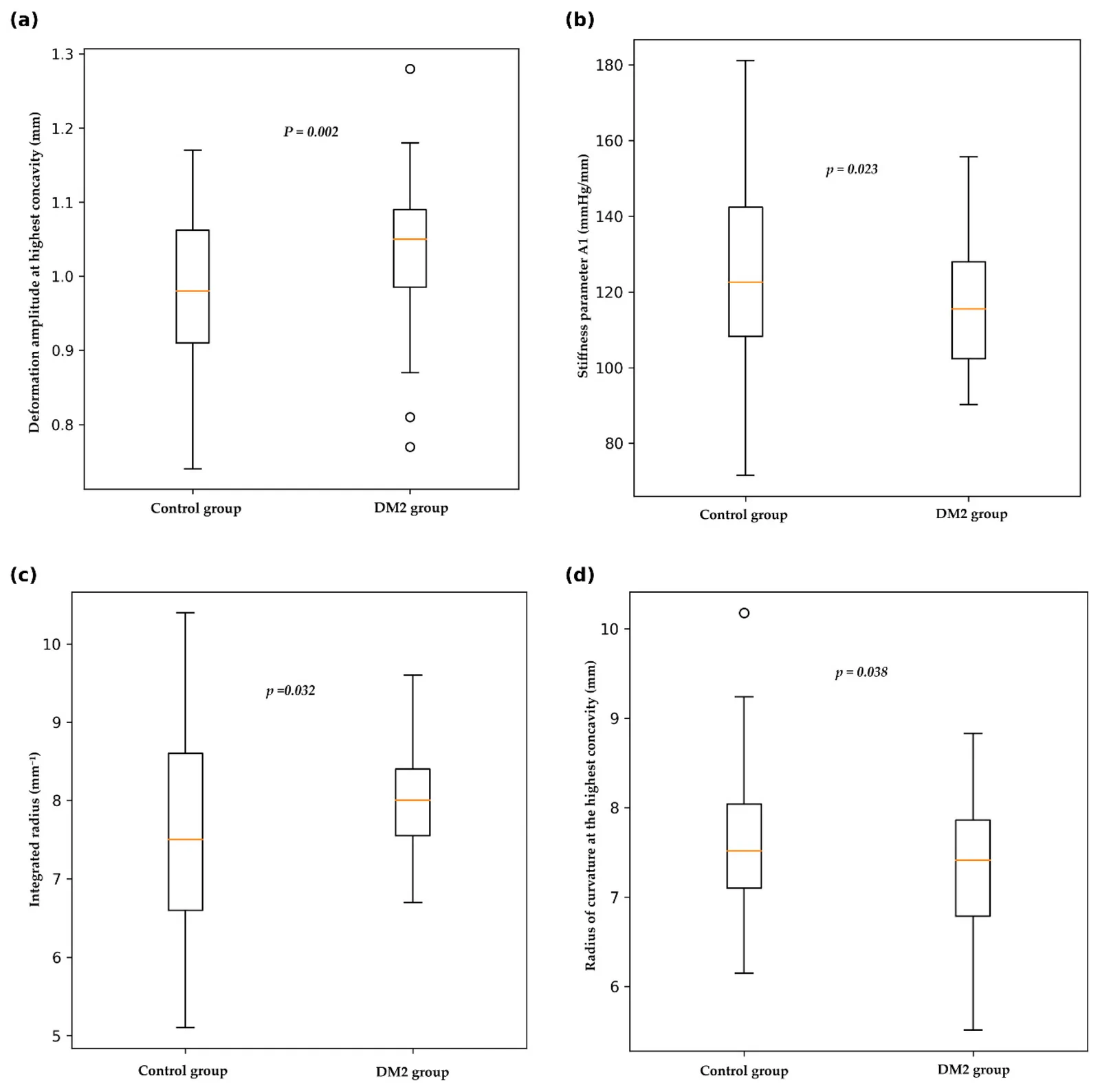
Corneal biomechanical parameters in the control and type 2 diabetes mellitus (DM2) groups: (**a**) deformation amplitude at highest concavity (DA HC); (**b**) stiffness parameter at first applanation (SP-A1); (**c**) integrated radius (IR); and (**d**) radius of curvature at highest concavity (RC). Box plots show the median and interquartile range, with whiskers and outliers displayed according to the box-plot convention. Corresponding *p*-values for between-group comparisons are shown within each panel.

The DA HC measurement, which represents the total displacement of the corneal apex from the start of deformation to the point of highest concavity, was significantly higher in the DM2 group (1.05, IQR 0.98–1.09) compared with the control group (0.98, IQR 0.91–1.06), with a Hodges–Lehmann median difference of 0.06 mm (95% CI 0.02 to 0.10; *p* = 0.002). The distribution of DA HC values in both groups is shown in Figure 1a.

Stiffness parameter at first applanation, an important biomechanical parameter defined as adjusted pressure minus IOP and divided by deflection amplitude at the first applanation, also differed statistically between the two groups. The median SP-A1 was significantly lower in the DM2 group (115.50 mmHg/mm, IQR 102.35–128.00) than in the control group (122.50 mmHg/mm, IQR 108.25–142.40), with a Hodges–Lehmann median difference of −8.40 mmHg/mm (95% CI −16.30 to −1.30; *p* = 0.023). The distribution of SP-A1 values in both groups is presented in Figure 1b.

Integrated radius, which represents the integrated area under the radius of the inversed curvature during the concave phase, was significantly higher in the DM2 group (8.00 mm^−1^, IQR 7.55–8.40) compared with the control group (7.50 mm^−1^, IQR 6.60–8.60), with a Hodges–Lehmann median difference of 0.40 mm^−1^ (95% CI 0.00 to 0.80; *p* = 0.032). IR comparison between groups is presented in Figure 1c.

Radius of curvature of the cornea at the highest concavity, which represents radius of curvature of a circle that fits to corneal concavity during maximum deformation, also showed statistically significant difference between groups. RC HC was significantly lower in the DM2 group (7.41 mm, IQR 6.79–7.86) than in the control group (7.52 mm, IQR 7.10–8.04), with a Hodges–Lehmann median difference of −0.31 mm (95% CI −0.59 to −0.02; *p* = 0.038). The distribution of RC across the study groups is illustrated in Figure 1d.

To account for potential confounding, multivariable linear regression analyses were performed for the biomechanical parameters that showed significant between-group differences in the unadjusted analyses, with adjustment for age, sex, CCT, and intraocular pressure. After adjustment, DM2 group status remained independently associated with higher DA HC (β = 0.0328 mm, 95% CI 0.0073–0.0582, *p* = 0.012). In contrast, the associations between DM2 status and RC HC (β = −0.2555 mm, 95% CI −0.5580 to 0.0470, *p* = 0.097), IR (β = 0.2585 mm^−1^, 95% CI −0.0093 to 0.5263, *p* = 0.058), and SP-A1 (β = −4.4622 mmHg/mm, 95% CI −8.9358 to 0.0114, *p* = 0.051) did not reach statistical significance after adjustment (Table 3).

An exploratory subgroup analysis comparing corneal biomechanical parameters between patients with type 2 DM with and without diabetic retinopathy is presented in Supplementary Table S1. 

### 3.2. Correlation Between Disease Duration and Corneal Biomechanical Parameters

Correlation analysis between diabetes duration and corneal biomechanical parameters showed statistically significant weak correlations for three parameters: A1L, PD, and DA HC. A weak negative correlation was observed between disease duration and A1L (Spearman’s ρ = −0.345, 95% CI −0.559 to −0.088; *p* = 0.010), indicating that longer disease duration was associated with a shorter length of flattened cornea at the first applanation. In contrast, weak positive correlations were observed for PD (ρ = 0.368, 95% CI 0.114 to 0.577; *p* = 0.006) and DA HC (ρ = 0.336, 95% CI 0.077 to 0.552; *p* = 0.012), suggesting that longer diabetes duration was associated with greater peak distance and increased deformation amplitude at highest concavity. No statistically significant correlations were observed for the remaining corneal biomechanical parameters (Table 4).

## 4. Discussion

The stroma accounts for approximately 90% of the corneal thickness and is the principal determinant of the cornea’s biomechanical properties. Its highly differentiated ultrastructure, with the special orientation, diameter, and separation of fibrillar collagen bundles and the regulatory role of other components of the extracellular matrix (proteoglycans and glycosaminoglycans), confer transparency and biomechanical behavior to the cornea [15]. In recent years, there has been growing interest in investigating the effects of systemic diseases on corneal biomechanics. Among these conditions, diabetes mellitus has received particular attention because of its potential to alter the structural and biomechanical properties of the cornea. Biomechanical corneal properties are crucial for maintaining shape and transparency of the cornea [9].

Based on the published literature to date, diabetes appears to influence corneal biomechanical properties, and there is a widely accepted concept that diabetes increases corneal stiffness through collagen cross-linking induced by AGEs [16]. Previous studies using the Ocular Response Analyzer (ORA), one of the first devices developed for in vivo assessment of corneal biomechanical behavior, reported alterations in patients with diabetes, with several studies demonstrating increased corneal hysteresis (CH) and corneal resistance factor (CRF) [16,17,18,19,20]. CH reflects the viscoelastic damping response of the cornea, whereas CRF is an empirically derived parameter related to the overall resistance of the cornea to deformation. Importantly, ORA-derived CH and CRF should not be considered directly equivalent to Corvis ST-derived parameters. Although both devices assess the corneal response to an air-puff stimulus, they use different measurement principles and characterize different aspects of corneal biomechanical behavior. Therefore, findings obtained with ORA provide complementary evidence regarding diabetes-related corneal biomechanical alterations but cannot be directly translated into corresponding Corvis ST parameters. Also, it was shown that CH and CRF correlated to hemoglobin A1c (HbA1c) and blood glucose concentration [16,17,18,19,20]. On the other hand, Corvis ST is a newer instrument integrated with an ultra-high-speed Scheimpflug camera, which enables the direct visualization of corneal movement during the application of a rapid air puff. Corvis ST measures biomechanical properties of the eye by recording the shape of the cornea as it becomes deformed in response to the application of an air pulse [21]. Nevertheless, in vivo assessment of corneal biomechanics has inherent limitations compared with ex vivo mechanical testing. Corvis ST provides an indirect characterization of biomechanical behavior rather than direct measurement of intrinsic tissue mechanical properties. In vivo measurements may also be influenced by factors such as intraocular pressure, corneal thickness, geometry, and the interaction of the cornea with surrounding ocular structures [22]. In contrast, ex vivo approaches, including tensile and inflation testing, allow more direct characterization of tissue mechanical properties under controlled experimental conditions and provide important insight into the stress–strain behavior of the cornea [22,23]. However, removal of the cornea from its physiological environment may alter tissue hydration, boundary conditions, and normal loading characteristics, thereby limiting direct extrapolation of ex vivo findings to the living eye [23]. Thus, in vivo and ex vivo approaches should be considered complementary rather than interchangeable, with Corvis ST providing clinically relevant information on the integrated biomechanical response of the cornea in its physiological ocular environment.

While many studies have investigated corneal biomechanics in diabetic patients using ORA, considerably fewer studies have evaluated corneal biomechanics in patients with diabetes mellitus using the Corvis ST. Although most published studies on Corvis ST suggest a trend toward increased corneal stiffness in diabetic eyes, the reported findings remain inconsistent, with substantial variability across individual biomechanical parameters [7,24,25]. Therefore, the aim of this study was to evaluate and compare Corvis ST-derived corneal biomechanical parameters between healthy individuals and patients with type 2 DM. Additionally, we analyzed the associations between disease duration and corneal biomechanical parameters.

In the current study, unadjusted comparisons showed significantly higher deformation amplitude at highest concavity and integrated radius values in diabetic eyes, whereas radius of curvature at highest concavity and stiffness parameter at first applanation were significantly lower. After adjustment for age, sex, central corneal thickness, and intraocular pressure, only increased DA HC remained independently associated with DM2 status. Additionally, longer disease duration was associated with significant, but weak, changes in DA HC, PD, and A1L, suggesting that prolonged diabetes may be accompanied by gradual alterations in corneal biomechanics.

DA HC is one of the most reliable corneal parameters obtained with the Corvis ST, and represents the maximal vertical displacement of the cornea during the air-puff induced deformation, at the point of highest concavity [26]. Among the analyzed parameters, DA HC exhibited the greatest between-group difference. The significantly higher DA HC values in the diabetic group suggest that the corneas of these patients were more deformable than those of healthy controls. Importantly, this association remained statistically significant after adjustment for age, sex, CCT, and intraocular pressure, suggesting that the observed difference in DA HC was not explained solely by these potential confounding factors. This result differs with the widely accepted hypothesis that diabetes increases corneal stiffness through AGE-mediated collagen cross-linking [15]. However, corneal biomechanical behavior in diabetic patients is highly complex as diabetes affects biomechanical properties through numerous mechanisms, including impaired hydration control, endothelial cell dysfunction, oxidative stress and ECM remodeling associated with chronic hyperglycemia [7]. All of these mechanisms may weaken the structural stability of the cornea and increase corneal deformability. Furthermore, unadjusted comparisons showed higher IR and lower SP-A1 values in diabetic eyes, which, together with increased DA HC, suggested a biomechanical profile consistent with greater corneal deformability [27]. However, unlike DA HC, the associations of IR and SP-A1 with DM2 did not remain statistically significant after multivariable adjustment, indicating that these findings were not independent of the covariates included in the model.

The fourth parameter demonstrating a statistically significant difference was the radius of curvature at highest concavity. As RC HC reflects the radius of the central cornea at the point of maximum concavity, lower values may indicate greater corneal deformability or reduced structural resistance. In agreement with the higher IR, increased DA HC, and lower SP-A1 values, diabetic eyes showed slightly lower RC HC values. SP-A1 has been defined as the ratio between the applied load and the resulting displacement of the cornea at first applanation, with higher SP-A1 values indicating a stiffer cornea [28]. Although SP-A1 was significantly lower in the DM2 group in the current study, the median values in both groups remained within the physiological range. Therefore, while the observed differences reached statistical significance, both for RC HC and SP-A1, their clinical relevance remains uncertain. These findings should be interpreted with caution, as they may reflect subtle biomechanical differences rather than clinically meaningful impairment. Moreover, neither RC HC nor SP-A1 remained significantly associated with DM2 status after adjustment for potential confounding factors, further supporting a cautious interpretation of these unadjusted differences.

Studies that investigated influence of diabetes on biomechanical parameters of the cornea had conflicting results. Pérez-Rico et al. in their study compared uncontrolled DM eyes with HbA1c ≥ 7%, controlled DM eyes with HbA1c < 7% and healthy eyes. Their results differed from the current study, since DA HC showed significantly lower values in uncontrolled DM eyes compared to the healthy control eyes. They associated their results with an abnormal HbA1c and poor blood glucose control, since the DA HC was inversely correlated with blood glucose concentration and HbA1c percentage. Their findings indicate that the elastic properties of the cornea may reflect the metabolic state of the patient and that diabetic patients with high HbA1c levels may show a significant decrease in elasticity of the corneal substance [24]. Additionally, results from Ohn et al. were in agreement with those of Pérez-Rico, reporting that A1V, DA HC, deflection amplitude maximum, whole eye movement, and IR were inversely correlated with HbA1c [25]. A potential explanation for the discrepancy between these findings and those of the present study may be differences in metabolic control among the studied populations. Previous evidence suggests that glycemic status may influence corneal biomechanical properties; however, HbA1c and blood glucose levels were not systematically available in our cohort. Therefore, the degree of glycemic control in our patients cannot be determined, and differences between our findings and those of previous studies cannot be attributed specifically to metabolic control. The observed biomechanical profile should consequently be interpreted as an association with the presence of type 2 diabetes in the studied cohort rather than as evidence of a specific hyperglycemia-related biomechanical mechanism. Future studies incorporating HbA1c and blood glucose measurements are needed to clarify the contribution of metabolic control to these findings.

Studies using ORA have also reported differences in corneal biomechanical behavior between diabetic and non-diabetic individuals. Goldich et al. found significantly higher CCT, CH, and CRF values in diabetic eyes than in non-diabetic eyes [29]. These ORA-derived findings indicate an altered viscoelastic response and greater overall resistance to deformation in diabetic corneas; however, they should not be interpreted as directly corresponding to the Corvis ST-derived measures of deformation or stiffness evaluated in the present study. Goldich et al. also emphasized that, given the marked heterogeneity of diabetes, differences between groups may have been disproportionately influenced by the most severely affected patients in the study group [29]. A study by Scheler et al. also reported elevated values of CH and CRF in patients with uncontrolled diabetes compared to those with controlled diabetes, further supporting the idea that disease severity may have the effect on corneal biomechanical changes [20]. Conversely, a study by Sahin et al., reported that CH was significantly lower in diabetic patients, whereas CRF was not significantly different from that of control subjects, indicating less damping ability in DM patients [30]. Kara et al. evaluated corneal biomechanical properties, specifically CH and CRF, in children with diabetes without diabetic retinopathy. They found no significant associations between CH or CRF and fasting blood glucose, HbA1c levels, disease duration, or age. Furthermore, no statistically significant differences in corneal biomechanics were observed between diabetic and healthy children [31]. These conflicting findings may primarily reflect heterogeneity among studies, including differences in study populations, sample size, disease duration, glycemic control, and methodological approaches, as well as variability in biomechanical measurements. In addition, the effects of diabetes on corneal biomechanics may be influenced by multiple biological mechanisms.

Current study additionally evaluated the correlation between disease duration and corneal biomechanical parameters in diabetic patients. A weak negative correlation was observed between disease duration and A1L, indicating that longer disease duration was associated with a shorter flattened corneal length at the time point of first applanation. This finding may suggest that corneal biomechanical properties differ according to disease duration, potentially reflecting duration-related alterations in corneal biomechanical behavior. On the other hand, disease duration showed weak positive correlations with PD and DA HC, suggesting that longer diabetes duration was associated with greater peak distance and increased deformation amplitude at the point of highest concavity, findings that may be consistent with increased corneal deformability. However, all observed correlations were weak and should therefore be interpreted with caution. Further studies incorporating additional clinical variables, such as glycemic control, diabetic retinopathy status, and longitudinal follow-up, are needed to better characterize the relationship between disease duration and corneal biomechanical changes.

This study contains certain limitations. Although several statistically significant differences were observed in the unadjusted analyses, only DA HC remained significantly associated with DM2 after multivariable adjustment. The relatively small sample size may have limited the statistical power of the adjusted analyses, particularly for detecting more subtle independent associations. Second, an important limitation is the absence of systematically collected measures of glycemic control, including HbA1c and blood glucose measurements. HbA1c is an established marker of long-term glycemic control and may be associated with alterations in corneal biomechanical properties; however, as an average measure of glycemia over the preceding months, it does not capture short-term glycemic variability or the magnitude and duration of individual hyperglycemic excursions. Continuous glucose monitoring (CGM)-derived metrics, particularly time in range (TIR) and time above range (TAR), could therefore provide complementary information on glycemic exposure and may allow a more detailed assessment of the relationship between hyperglycemia and corneal biomechanical alterations. The absence of both conventional glycemic markers and CGM-derived data prevented us from examining whether the observed corneal biomechanical differences were related to the degree or temporal pattern of hyperglycemic exposure. Accordingly, the observed between-group differences cannot be specifically attributed to the degree of hyperglycemia or metabolic control, and should be interpreted as associations observed in patients with type 2 diabetes rather than evidence of a specific diabetes-related biomechanical mechanism. Furthermore, diabetes was excluded in the control group based on medical history and available medical records rather than systematic biochemical screening with fasting plasma glucose and/or HbA1c. Therefore, the possibility of unrecognized disturbances in glucose metabolism among control participants cannot be completely excluded. In addition, detailed information regarding duration of diabetes treatment and relevant systemic comorbidities, particularly arterial hypertension and dyslipidemia, was not systematically collected for all participants. These conditions are common in patients with DM type 2 and may differ in prevalence between diabetic and non-diabetic populations. As they could potentially act as systemic confounding factors, the inability to account for their possible influence on corneal biomechanical measurements represents an additional limitation of the present study.

Finally, the diabetic cohort had a relatively short median disease duration of approximately seven years, and most participants did not have diabetic retinopathy. Consequently, our findings may predominantly reflect corneal biomechanical characteristics in relatively earlier stages of DM type 2, and it remains uncertain whether patients with longer disease duration, poorer metabolic control, or more advanced diabetic retinopathy would exhibit more pronounced biomechanical alterations. Therefore, these findings should be generalized with caution to patients with more advanced diabetes, longer disease duration, or poor glycemic control.

Future prospective studies with larger cohorts, systematic assessment of conventional glycemic markers such as HbA1c and fasting glucose, and, where feasible, incorporation of CGM-derived metrics such as TIR and TAR, together with comprehensive documentation of systemic comorbidities and inclusion of patients across a broader spectrum of diabetes duration and severity, are warranted to further clarify the relationship between glycemic exposure and corneal biomechanical alterations in type 2 diabetes.

## 5. Conclusions

This study showed that patients with type 2 diabetes exhibited differences in corneal biomechanical parameters suggestive of greater corneal deformability, with increased DA HC and IR values and lower RC HC and SP-A1 values observed in unadjusted comparisons. After adjustment for age, sex, CCT, and intraocular pressure, increased DA HC remained independently associated with DM2 status, whereas the associations with IR, RC HC, and SP-A1 were attenuated and no longer statistically significant. These findings were observed in a cohort predominantly comprising patients with a relatively short disease duration, treated mostly with oral antidiabetic agents, with more than half of the patients showing no evidence of diabetic retinopathy, thus primarily reflecting patients at relatively earlier stages of type 2 diabetes. The observed between-group differences were small, and several parameters remained within the physiological range; therefore, their clinical relevance should be interpreted with caution. Overall, these findings highlight the variability and complexity of corneal biomechanical alterations associated with diabetes and suggest that diabetes-related biomechanical changes may not uniformly manifest as increased corneal stiffness. However, because metabolic control was not assessed, the observed differences cannot be specifically attributed to chronic hyperglycemia or other diabetes-related metabolic mechanisms. Further prospective studies incorporating glycemic control, diabetic retinopathy severity, and longer disease duration are warranted to better characterize the biomechanical changes associated with diabetes.

## Figures and Tables

**Table 1:** Baseline demographic and clinical characteristics.

		DM2 Group (*n* = 55)	Control Group (*n* = 60)	*p* Value
Age [median (IQR)]		63 (57–68)	56 (54–59)	<0.0001
Gender	Male	31	23	0.053
	Female	24	37	
Disease duration (years) Median (IQR)		7 (4–15)	-	-
Therapy type	Oral antidiabetic drugs	39	-	-
	Insulin	16		
Presence of diabetic retinopathy	Yes	24	-	-
	No	31		

DM2—diabetes mellitus type 2, IQR—interquartile range.

**Table 2:** Unadjusted comparison of pachymetric and Corvis ST biomechanical parameters between the type 2 diabetes mellitus and control groups.

Parameter	DM2 Group (*n* = 55) Median (IQR)	Control Group (*n* = 60) Median (IQR)	Hodges–Lehmann Difference (95% CI)	*p*-Value
CCT (µm)	551.0 (519.9–578.0)	562.0 (525.0–583.0)	−9.0 (−25.0 to 6.0)	0.243
SSI	1.26 (1.16–1.40)	1.31 (1.21–1.42)	−0.030 (−0.100 to 0.040)	0.305
CBI	0.30 (0.18–0.58)	0.29 (0.07–0.59)	0.030 (−0.070 to 0.130)	0.396
BGF	0.23 (0.12–0.35)	0.18 (0.10–0.27)	0.040 (−0.010 to 0.100)	0.098
A1L (mm)	2.26 (2.00–2.61)	2.34 (2.07–2.49)	−0.010 (−0.140 to 0.130)	0.922
A1V (m/s)	0.14 (0.11–0.15)	0.12 (0.11–0.14)	0.010 (0.000 to 0.020)	0.151
A1T (ms)	7.12 (6.91–7.42)	7.25 (7.05–7.57)	−0.110 (−0.250 to 0.030)	0.148
A2L (mm)	1.95 (1.87–2.09)	1.96 (1.87–2.08)	−0.010 (−0.080 to 0.070)	0.790
A2V (m/s)	−0.25 (−0.27–−0.23)	−0.24 (−0.25–−0.22)	−0.010 (−0.020 to 0.000)	0.080
A2T (ms)	21.53 (21.28–21.70)	21.44 (21.04–21.70)	0.080 (−0.090 to 0.250)	0.304
RC HC (mm)	7.41 (6.79–7.86)	7.52 (7.10–8.04)	−0.310 (−0.590 to −0.020)	0.038
PD (mm)	4.75 (4.58–4.93)	4.68 (4.48–4.86)	0.050 (−0.050 to 0.170)	0.317
DA HC (mm)	1.05 (0.98–1.09)	0.98 (0.91–1.06)	0.06 (0.02 to 0.10)	0.002
IOPnct (mmHg)	16.50 (15.0–18.0)	16.75 (15.3–18.2)	−0.50 (−1.50 to 0.50)	0.166
bIOP (mmHg)	15.00 (14.0–16.8)	15.85 (14.6–17.3)	−0.70 (−1.80 to 0.40)	0.233
DA ratio	4.2 (4.00–4.40)	4.0 (3.77–4.40)	0.10 (0.00 to 0.30)	0.074
IR (mm^−1^)	8.00 (7.55–8.40)	7.50 (6.60–8.60)	0.40 (0.00 to 0.80)	0.032
ARTh (µm)	485.6 (410.0–559.0)	445.7 (382.0–512.0)	11.1 (−34.0 to 55.7)	0.654
SP-A1 (mmHg/mm)	115.50 (102.35–128.0)	122.50 (108.25–142.40)	−8.40 (−16.30 to −1.30)	0.023

DM2—diabetes mellitus type 2, IQR—interquartile range, CI—confidence interval, CCT—central corneal thickness, SSI—Stress–Strain Index, CBI—Corvis Biomechanical Index, BGF—biomechanical glaucoma factor, A1L—length of flattened cornea at the first applanation, A1V—velocity at the first applanation, A1T—time taken to reach the first applanation, A2L—length of flattened cornea at the second applanation, A2V—velocity at the second applanation, A2T—time taken to reach the second applanation, RC HC—radius of curvature of the cornea at highest concavity, PD—peak distance at maximal concavity, DA HC—deformation amplitude at highest concavity, IOPnct—non-contact tonometer measured IOP, bIOP—biomechanically corrected intraocular pressure, DA ratio—deformation amplitude ratio, IR—integrated radius, ARTh—Ambrósio relational thickness to the horizontal profile, SP-A1—stiffness parameter at first applanation. Hodges–Lehmann estimates represent the difference between the DM2 and control groups (DM2—control).

**Table 3:** Multivariable-adjusted association between type 2 diabetes mellitus and selected corneal biomechanical parameters.

Outcome	Adjusted β for DM2 Group	95% CI	*p*-Value
DA HC (mm)	0.0328	0.0073 to 0.0582	0.012
RC HC (mm)	−0.2555	−0.5580 to 0.0470	0.097
IR (mm^−1^)	0.2585	−0.0093 to 0.5263	0.058
SP-A1 (mmHg/mm)	−4.4622	−8.9358 to 0.0114	0.051

β represents the adjusted regression coefficient for the DM2 group compared with the control group. All models were adjusted for age, sex, central corneal thickness and intraocular pressure. DM2—diabetes mellitus type 2, CI—confidence interval, DA HC—deformation amplitude at highest concavity, RC HC—radius of curvature at highest concavity, IR—integrated radius, SP-A1—stiffness parameter at first applanation.

**Table 4:** Correlation between disease duration and corneal biomechanical parameters in diabetic eyes.

Parameter	Spearman’s Rho	95% CI	*p*-Value
CCT (µm)	−0.039	−0.301 to 0.229	0.779
SSI	−0.184	−0.428 to 0.085	0.179
CBI	0.068	−0.201 to 0.328	0.620
BGF	0.077	−0.192 to 0.336	0.575
A1L (mm)	−0.345	−0.559 to −0.088	0.01
A1V (m/s)	0.048	−0.220 to 0.309	0.729
A1T (ms)	−0.223	−0.461 to 0.045	0.102
A2L (mm)	−0.076	−0.335 to 0.193	0.579
A2V (m/s)	−0.206	−0.447 to 0.063	0.132
A2T (ms)	−0.032	−0.295 to 0.235	0.816
RC HC (mm)	0.094	−0.176 to 0.350	0.496
PD (mm)	0.368	0.114 to 0.577	0.006
DA HC (mm)	0.336	0.077 to 0.552	0.012
IOPnct (mmHg)	−0.148	−0.398 to 0.122	0.279
bIOP (mmHg)	−0.137	−0.389 to 0.133	0.317
DA ratio	−0.058	−0.319 to 0.210	0.673
IR (mm^−1^)	−0.151	−0.400 to 0.119	0.271
ARTh (µm)	−0.069	−0.329 to 0.199	0.613
SP-A1 (mmHg/mm)	−0.042	−0.304 to 0.226	0.763

CCT—central corneal thickness, SSI—Stress–Strain Index, CBI—Corvis Biomechanical Index, BGF—biomechanical glaucoma factor, A1L—length of flattened cornea at the first applanation, A1V—velocity at the first applanation, A1T—time taken to reach the first applanation, A2L—length of flattened cornea at the second applanation, A2V—velocity at the second applanation, A2T—time taken to reach the second applanation, RC HC—radius of curvature of the cornea at highest concavity, PD—peak distance at maximal concavity, DA HC—deformation amplitude at highest concavity, IOPnct—non-contact tonometer measured IOP, bIOP—biomechanically corrected intraocular pressure, DA ratio—deformation amplitude ratio, IR—integrated radius, ARTh—Ambrósio relational thickness to the horizontal profile, SP-A1—stiffness parameter at first applanation, CI—confidence interval.

## Data Availability

The data presented in this study are available upon request from the corresponding author. The data are not publicly available due to privacy and ethical restrictions.

## Supplementary Materials

The following supporting information can be downloaded at: https://www.mdpi.com/article/10.3390/medicina62101928/s1, Table S1: Comparison of corneal biomechanical parameters between patients with type 2 diabetes mellitus with and without diabetic retinopathy.

## Abbreviations

The following abbreviations are used in this manuscript:

DM	Diabetes mellitus
CCT	Central corneal thickness
AGEs	Advanced glycation end products
ECM	Extracellular matrix
SSI	Stress–strain index
CBI	Corvis biomechanical index
BGF	Biomechanical glaucoma factor
A1L	Length of flattened cornea at the first applanation
A1V	Velocity at the first applanation
A2T	Time taken to reach the first applanation
A2L	Length of flattened cornea at the second applanation
A2V	Velocity at the second applanation
A2T	Time taken to reach the second applanation
RC HC	Radius of curvature at highest concavity
PD	Peak distance
DA HC	Deformation amplitude at highest concavity
IOPnct	Non-contact tonometer measured intraocular pressure
bIOP	Biomechanically corrected intraocular pressure
DA ratio	Deformation amplitude ratio
IR	Integrated radius
ARTh	Ambrósio relational thickness to the horizontal profile
SP-A1	Stiffness parameter at first applanation
ORA	Ocular Response Analyser

## References

[1] International Diabetes Federation (2025). IDF Diabetes Atlas.

[2] Romero-Aroca P. (2022). Ocular Complications of Diabetes and Therapeutic Approaches. J. Clin. Med..

[3] Studnička J., Němčanský J., Vysloužilová D., Ernest J., Němec P. (2023). Diabetic Retinopathy—Diagnostics and Treatment Guideline. Cesk Slov. Oftalmol..

[4] Uzunoglu A., Valenzuela-Fuenzalida J.J., Morales-Calderón K., Aguilar-Aguirre I., Bruna-Mejias A., Nova-Baeza P., Orellana-Donoso M., Oyanedel-Amaro G., Suazo-Santibañez A., Sanchis-Gimeno J.A. (2025). The Effect of Diabetes Mellitus on Central Corneal Thickness Values: A Systematic Review and Meta-Analysis. Int. J. Mol. Sci..

[5] Sayin N., Kara N., Pekel G. (2015). Ocular complications of diabetes mellitus. World J. Diabetes.

[6] Ljubimov A.V. (2017). Diabetic complications in the cornea. Vis. Res..

[7] Ramm L., Herber R., Terai N., Pillunat L.E. (2019). Factors influencing corneal biomechanics in diabetes mellitus. Investig. Ophthalmol. Vis. Sci..

[8] Wang X., McAlinden C., Zhang H., Yan J., Wang D., Wei W., Mi S. (2021). Assessment of corneal biomechanics, tonometry and pachymetry with the Corvis ST in myopia. Sci. Rep..

[9] Komninou M.A., Seiler T.G., Enzmann V. (2024). Corneal biomechanics and diagnostics: A review. Int. Ophthalmol..

[10] Garcia-Porta N., Fernandes P., Queiros A., Salgado-Borges J., Parafita-Mato M., González-Méijome J.M. (2014). Corneal Biomechanical Properties in Different Ocular Conditions and New Measurement Techniques. ISRN Ophthalmol..

[11] Wang X., Xu G., Wang W., Wang J., Chen L., He M., Chen Z. (2020). Changes in corneal biomechanics in patients with diabetes mellitus: A systematic review and meta-analysis. Acta Diabetol..

[12] Wilkinson C.P., Ferris F.L., Klein R.E., Lee P.P., Agardh C.D., Davis M., Dills D., Kampik A., Pararajasegaram R., Verdaguer J.T. (2003). Proposed international clinical diabetic retinopathy and diabetic macular edema disease severity scales. Ophthalmology.

[13] Yu A.-Y., Shao H., Pan A., Wang Q., Huang Z., Song B., McAlinden C., Huang J., Chen S. (2020). Corneal biomechanical properties in myopic eyes evaluated via Scheimpflug imaging. BMC Ophthalmol..

[14] Yii F., Strang N., Bernabeu M.O., Dhillon B., MacGillivray T. (2024). Corneal biomechanics are not exclusively compromised in high myopia. Ophthalmic Physiol. Opt..

[15] del Buey M.A., Casas P., Caramello C., López N., de la Rica M., Subirón A.B., Lanchares E., Huerva V., Grzybowski A., Ascaso F.J. (2019). An Update on Corneal Biomechanics and Architecture in Diabetes. J. Ophthalmol..

[16] Kotecha A., Oddone F., Sinapis C., Elsheikh A., Sinapis D., Sinapis A., Garway-Heath D.F. (2010). Corneal biomechanical characteristics in patients with diabetes mellitus. J. Cataract. Refract. Surg..

[17] Luce D.A. (2005). Determining in vivo biomechanical properties of the cornea with an ocular response analyzer. J. Cataract. Refract. Surg..

[18] Dupps W.J., Wilson S.E. (2006). Biomechanics and wound healing in the cornea. Exp. Eye Res..

[19] Roberts C. (2000). The cornea is not a piece of plastic. J. Refract. Surg..

[20] Scheler A., Spoerl E., Boehm A.G. (2012). Effect of diabetes mellitus on corneal biomechanics and measurement of intraocular pressure. Acta Ophthalmol..

[21] Boszczyk A., Kasprzak H., Przeździecka-Dołyk J. (2022). Novel Method of Measuring Corneal Viscoelasticity Using the Corvis ST Tonometer. J. Clin. Med..

[22] Yuhas P.T., Roberts C.J. (2023). Clinical Ocular Biomechanics: Where Are We after 20 Years of Progress?. Curr. Eye Res..

[23] Mascolini M.V., Toniolo I., Carniel E.L., Fontanella C.G. (2024). Ex vivo, in vivo and in silico studies of corneal biomechanics: A systematic review. Phys. Eng. Sci. Med..

[24] Pérez-Rico C., Gutiérrez-Ortíz C., González-Mesa A., Zandueta A.M., Moreno-Salgueiro A., Germain F. (2015). Effect of diabetes mellitus on Corvis ST measurement process. Acta Ophthalmol..

[25] Ohn K., Noh Y.H., Moon J.I., Jung Y. (2022). Measurement of corneal biomechanical properties in diabetes mellitus using the Corvis ST. Medicine.

[26] Vinciguerra R., Ambrósio R., Elsheikh A., Roberts C.J., Lopes B., Morenghi E., Azzolini C., Vinciguerra P. (2016). Detection of Keratoconus with a New Biomechanical Index. J. Refract. Surg..

[27] Vinciguerra R., Elsheikh A., Roberts C.J., Ambrósio R., Kang D.S., Lopes B.T., Morenghi E., Azzolini C., Vinciguerra P. (2016). Influence of Pachymetry and Intraocular Pressure on Dynamic Corneal Response Parameters in Healthy Patients. J. Refract. Surg..

[28] Aoki S., Asaoka R., Azuma K., Kitamoto K., Ueda K., Inoue T., Obata R. (2024). Biomechanical properties measured with dynamic Scheimpflug analyzer in central serous chorioretinopathy. Graefes Arch. Clin. Exp. Ophthalmol..

[29] Goldich Y., Barkana Y., Gerber Y., Rasko A., Morad Y., Harstein M., Avni I., Zadok D. (2009). Effect of diabetes mellitus on biomechanical parameters of the cornea. J. Cataract. Refract. Surg..

[30] Sahin A., Bayer A., Ozge G., Mumcuoğlu T. (2009). Corneal biomechanical changes in diabetes mellitus and their influence on intraocular pressure measurements. Investig. Ophthalmol. Vis. Sci..

[31] Kara N., Yildirim Y., Univar T., Kontbay T. (2013). Corneal biomechanical properties in children with diabetes mellitus. Eur. J. Ophthalmol..

