# Supplementary material for: Corvis ST Assessment of Corneal Biomechanical Properties in Patients with Type 2 Diabetes Mellitus
Source: Medicina (Kaunas). 2026 Oct 7;62(10):1928. doi: 10.3390/medicina62101928 (PMC13706947; doi:10.3390/medicina62101928)
Supplement: Supplementary file 1 [file medicina-62-01928-s001.zip › medicina-4499498-supplementary.pdf]

Supplementary Table S1. Comparison of corneal biomechanical parameters between patients with type 2 diabetes mellitus with and without diabetic retinopathy.

Variable	**No DR (n = 31)** Median (IQR)	**DR (n = 24)** Median (IQR)	**p-value**	**FDR-adjusted p-value**
CCT (μm)	556.0 (530.8–568.0)	520.5 (507.5–589.5)	0.678	0.859
SSI	1.26 (1.18–1.46)	1.25 (1.15–1.40)	0.415	0.859
CBI	0.300 (0.130–0.518)	0.315 (0.225–0.635)	0.391	0.859
BGF	0.230 (0.140–0.350)	0.205 (0.110–0.330)	0.513	0.859
A1L (mm)	2.350 (2.185–2.610)	2.085 (1.865–2.630)	0.075	0.580
A1V (m/s)	0.140 (0.120–0.148)	0.135 (0.110–0.150)	0.586	0.859
A1T (ms)	7.110 (6.988–7.320)	7.135 (6.810–7.580)	0.926	0.977
A2L (mm)	1.940 (1.880–2.173)	1.955 (1.705–2.050)	0.513	0.859
A2V (m/s)	−0.250 (−0.268 to −0.223)	−0.245 (−0.275 to −0.230)	0.626	0.859
A2T (ms)	21.530 (21.370–21.645)	21.435 (21.090–21.765)	0.445	0.859
RC HC (mm)	7.130 (6.730–7.870)	7.465 (6.815–7.855)	0.671	0.859
PD (mm)	4.670 (4.490–4.885)	4.900 (4.610–4.990)	0.118	0.580
DA HC (mm)	1.040 (0.965–1.088)	1.075 (0.990–1.125)	0.174	0.661
IOPnct (mmHg)	16.0 (15.13–18.00)	16.5 (14.00–20.00)	0.825	0.922
bIOP (mmHg)	15.00 (13.93–16.20)	14.75 (12.95–19.05)	0.993	0.993
DA ratio	4.30 (4.00–4.40)	4.10 (3.95–4.30)	0.122	0.580
IR (mm^{-1})	8.20 (7.63–8.68)	7.80 (7.30–8.15)	**0.036**	0.580
ARTh (μm)	491.5 (432.3–590.3)	480.7 (385.2–539.9)	0.301	0.859
SP-A1 (mmHg/mm)	115.70 (102.85–128.40)	114.05 (100.40–128.85)	0.767	0.911

Between-group comparisons were performed using the Mann–Whitney U test. To account for multiple comparisons, p-values were adjusted using the Benjamini–Hochberg false discovery rate (FDR) procedure. Statistical significance was defined as an FDR-adjusted p-value < 0.05. DR, diabetic retinopathy. IQR – interquartile range, CCT – central corneal thickness, SSI - Stress-Strain Index, CBI - Corvis Biomechanical Index, BGF - biomechanical glaucoma factor, A1L - length of flattened cornea at the first applanation, A1V – velocity at the first applanation, A1T – time taken to reach the first applanation, A2L - length of flattened cornea at the second applanation, A2V – velocity at the second applanation, A2T – time taken to reach the second applanation, RC HC - radius of curvature of the cornea at highest concavity, PD - peak distance at maximal concavity, DA HC - deformation amplitude at highest concavity, IOPnct - non-contact tonometer measured IOP, bIOP - biomechanically corrected intraocular pressure, DA ratio - deformation amplitude ratio, IR - integrated radius, ARTh - Ambrósio relational thickness to the horizontal profile, SP-A1 - stiffness parameter at first applanation.
